# Enhancing LncRNA-miRNA interaction prediction with multimodal contrastive representation learning

**DOI:** 10.1093/bib/bbaf281

**Published:** 2025-06-17

**Authors:** Zhixia Teng, Zhaowen Tian, Murong Zhou, Guohua Wang, Zhen Tian, Yuming Zhao

**Affiliations:** College of Computer and Control Engineering, Northeast Forestry University, 150040, Harbin, China; College of Computer and Control Engineering, Northeast Forestry University, 150040, Harbin, China; College of Computer and Control Engineering, Northeast Forestry University, 150040, Harbin, China; College of Computer and Control Engineering, Northeast Forestry University, 150040, Harbin, China; School of Computer and Artificial Intelligence, Zhengzhou University, 450001, Zhengzhou, China; Yangtze Delta Region Institute (Quzhou), University of Electronic Science and Technology of China, 324000, Quzhou, China; College of Computer and Control Engineering, Northeast Forestry University, 150040, Harbin, China

**Keywords:** lncRNA-miRNA interaction, multimodal features, contrastive learning, representation learning, Transformer

## Abstract

Interactions between long non-coding RNAs (lncRNAs) and microRNAs (miRNAs) play an important role in the development of complex human diseases by collaboratively regulating gene transcription and expression. Therefore, identifying lncRNA-miRNA interactions (LMIs) is essential for diagnosing and treating complex human diseases. Because identifying LMIs with wet experiments is time-consuming and labor-intensive, some computational methods have been developed to infer LMIs. However, these approaches excel at utilizing single-modal information but struggle to integrate multimodal data from lncRNAs and miRNAs, which is essential for uncovering complex patterns in LMIs, ultimately limiting their performance. Therefore, this article proposes a novel multimodal contrastive representation learning model (MCRLMI) for LMI predictions. The model fully integrates multi-source similarity information and sequence encodings of lncRNAs and miRNAs. It leverages a graph convolutional network (GCN) and a Transformer to capture local neighborhood structural features and long-distance dependencies, respectively, enabling the collaborative modeling of structural and semantic information. Subsequently, to effectively integrate multimodal characteristics with encoded information, a multichannel attention mechanism and contrastive learning are introduced to fuse the extracted features. Finally, a Kolmogorov–Arnold Network (KAN) is trained with the optimized embeddings to predict LMIs. Extensive experiments show that the proposed MCRLMI consistently outperforms existing methods. Moreover, case studies further validate the potential of MCRLMI to identify novel LMIs in practical applications.

## Introduction

Non-coding RNAs (ncRNAs) [1], particularly lncRNAs and miRNAs, play an indispensable role in many life processes [2]. LncRNAs, which typically exceed 200 nucleotides in length, are involved in epigenetic regulation and transcriptional control [3, 4], making them important regulatory factors in the human genome [5]. MiRNAs, with sequences approximately 22 nucleotides in length, regulate gene expression at the post-transcriptional level in animals and plants [6, 7]. Numerous studies have demonstrated that lncRNA and miRNA interactions may significantly impact many biological activities. For example, Tsang *et al.* found that miR-125b can downregulate the expression of HOTTIP, an oncogenic lncRNA, involved in regulating hepatocellular carcinoma [8]. Numerous experiments showed that the lncRNA HOTAIR interacts with miRNAs, influencing the progression of cancers [9]. Therefore, exploring unknown interactions between lncRNAs and miRNAs not only helps us better understand the functional expressions of lncRNAs and miRNAs [5] but also provides new insights into the pathogenesis of human complex diseases [10].

Previous studies depended on traditional biological experiments to investigate unknown LMIs [5]. However, such experiments are costly and time-intensive, making it hard to satisfy the rapidly increasing demand for predicting LMIs [11]. Therefore, there is an urgent need for computational tools to discover novel LMIs efficiently [10]. With the rapid development of computer technology, especially the widespread application of machine learning and deep learning methods [12] in various fields of bioinformatics, several computational methods have been utilized to predict interactions between lncRNAs and miRNAs. Based on the input, the existing computational LMI prediction approaches can be roughly divided into sequence-based and network-based methods [13].

Sequence-based methods typically rely on the sequence information of lncRNAs and miRNAs to predict LMIs. In the methods, two feature encoding strategies are usually used to analyze sequences of lncRNAs and miRNAs. The first involves the compositional and statistical properties of the sequences, such as k-mers [14], the composition transition distribution (CTD) [15], the GC content, and the base counts. The second refers to the encoding methods, such as one-hot encoding, doc2vec, and role2vec, which convert sequences into feature vectors. These features are then fed into deep-learning models to predict LMIs. For example, Yang *et al.* [17] extracted four types of features from sequences, including k-mers, CTD, doc2vec [16], and role2vec [18], followed by a convolutional neural network (CNN) for deep feature extraction to screen LMIs. Kang *et al.* [19] proposed a framework that integrates deep and shallow feature extraction strategies for RNA sequence classification. The deep learning branch utilizes a CNN-BiGRU network to capture high-level sequence patterns automatically. In contrast, the shallow learning branch derives handcrafted features, including k-mer frequency, GC content, base pair counts, and minimum free energy (MFE), used to train a random forest classifier. Zhang *et al.* [20] employed CNN to extract functional features and adopted independent recurrent neural networks (IndRNNs) to learn sequence dependencies, finally using fully connected layers to discover LMIs. Kang *et al.* [21] extracted k-mer, g-gap, and secondary structure features from RNA sequences and fused them into a unified representation. Then, by integrating complex features, multi-scale convolutional long short-term memory networks, and attention mechanisms, they enhanced the sample information at the feature, scale, and model levels, respectively. Finally, an ensemble deep learning model was built based on a greedy fuzzy decision method, significantly improving efficiency. Kang *et al.* [22] extracted multiple base features from RNA data. They constructed complex features using an arithmetic-level method and further enhanced the sample information through an arithmetic mean strategy. At the same time, three feature ranking methods were integrated for feature selection, enabling the adaptive retention of important features and removal of redundant ones. Yu *et al.* [11] extracted k-mers and input them into RNA2vec, which can convert RNA sequences into vector representations. CNN and Bi-GRU were then adopted to learn features, and finally, trained an LMI prediction model. Wang *et al.* [23] leveraged a method similar to Yu *et al.* [11], employing doc2vec for pretraining followed by graph neural network (GNN) for fine-tuning. Chen *et al.* [24] proposed a differential encoding strategy tailored to the sequence differences between miRNA and lncRNA. And they integrated a hybrid feature mining network with an ensemble module to predict LMIs. Sequence-based methods can effectively leverage sequence information to extract advanced features through deep learning techniques. However, they still have some limitations. For instance, while compositional and statistical features effectively capture local discrete information from unimodal sequences, they often overlook crucial long-range dependencies between the underlying factors in the sequences. Additionally, One-hot encoding at the nucleotide level may introduce excessive redundant features, thereby hindering the effective capture of the underlying structural or functional patterns in RNA sequences.

Network-based approaches often transform the LMI prediction task as a fully connected prediction problem within a heterogeneous lncRNA-miRNA network. A bipartite lncRNA-miRNA network is constructed using sequence information, expression profiles, and known LMIs. Then, statistical methods, machine learning, or GNN approaches are applied to extract features from the bipartite network to calculate LMI scores, thereby identifying potential LMIs. For example, Wang *et al.* [5] constructed a heterogeneous network using sequence similarity, Gaussian interaction profile (GIP) kernel similarity of lncRNAs and miRNAs, and known LMIs. They first employed a GCN to generate initial node embeddings in the heterogeneous network, then refined these embeddings using a conditional random field (CRF), and finally utilized a decoder to analyze the embeddings and compute the scores of candidate LMIs. Similarly, Zhang *et al.* [25] explored a network distance analysis method to estimate scores of candidate LMIs based on the lncRNA-miRNA network. Tian *et al.* [26] leveraged second-order graph convolution (SOGCN) and various graph embedding techniques to obtain feature representations of lncRNAs and miRNAs, followed by matrix completion to predict novel LMIs. Zhao *et al.* [27] first encoded k-mer sequence fragments of lncRNAs and miRNAs as their initial features by word2vec. They then explored the GCN and the Rotation Forest (RoF) to identify potential LMIs in the lncRNA-miRNA network. Network-based methods can effectively utilize the network topological information to predict LMIs. However, these methods often construct heterogeneous networks by simply integrating multimodal heterogeneous data, which limits their ability to capture key information from different modal data and fully exploit cross-modal complementarity to characterize LMIs effectively.

Sequence-based methods focus on extracting sequence features of lncRNA and miRNA, while network-based methods leverage the network topology to reveal the interaction relationships between lncRNA and miRNA.These methods perform well in utilizing single-modal information but struggle to fuse multimodal data, which is crucial for capturing unique representations of complex LMI patterns. To address the challenges described above, this study proposes a novel multimodal contrastive representation learning method called MCRLMI for predicting potential LMIs. We first construct similarity matrices based on multimodal data and utilize GCN to capture local neighborhood structural features. Then, several encoding matrices at different scales $\text{k}$-mers are built and fed into the Transformer to capture long-distance dependency features. Next, a multichannel attention mechanism is employed to learn discriminative features of lncRNAs and miRNAs from different modalities, followed by contrastive learning to integrate local neighborhood structural features and long-distance dependency features. Finally, a Kolmogorov–Arnold Network (KAN) is utilized to effectively fuse the embeddings of lncRNAs and miRNAs for accurate LMI identification. In general, the main contributions of the proposed MCRLMI can be summarized as follows:

(1) MCRLMI effectively captures long-distance dependencies and local neighborhood structural features of lncRNAs and miRNAs across multimodal data.
(2) MCRLMI learns representative and discriminative features through contrastive learning combined with a multichannel attention mechanism across different modalities.
(3) MCRLMI accurately characterizes LMIs through integrating lncRNAs and miRNAs features with a dynamic interactive edge-function mapping mechanism of KAN.
(4) Extensive experiments demonstrate that MCRLMI surpasses existing approaches, and case studies further highlight its effectiveness in identifying potential LMIs in practice.

## Method

**Figure 1:**
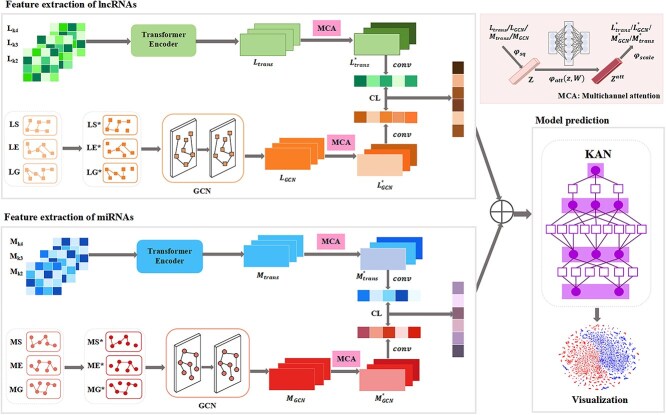
The framework of MCRLMI. Firstly, similarity and encoding matrices are derived from multimodal data of lncRNAs and miRNAs. Secondly, the similarity matrices are processed by the GCN while the positional encoding matrices are analyzed by the Transformer. Next, a multichannel attention mechanism is applied to capture representative features. Then, contrastive learning is employed to learn the discriminative features. Finally, KAN is used to predict potential LMIs. In this figure, CL refers to contrastive learning, and MCA stands for the multichannel attention mechanism.

The framework is shown in Fig. 1. First, we construct similarity matrices from multimodal data and then apply the nearest-neighbor graph to refine these matrices. Then, GCN is employed to extract local neighborhood structural features of lncRNAs and miRNAs from these matrices. Meanwhile, the sequences of lncRNAs and miRNAs are characterized by a one-hot encoding method at multiscale k-mers. Then, their sequence encodings are input into the Transformer encoder to capture the long-distance dependencies features of lncRNAs and miRNAs. After that, a multichannel attention mechanism is adopted to extract representative features from the multimodal matrices, while contrastive learning is utilized to obtain discriminative features further. Finally, the learned representations of lncRNAs and miRNAs are input into KAN for LMI predictions.

### Local neighborhood structural features

#### The similarity calculation of lncRNAs and miRNAs

To obtain multimodal similarity matrices of lncRNAs and miRNAs, three types of their similarities: sequence similarity, expression profile similarity, and GIP kernel similarity, are calculated in this article. Specifically, the sequence similarities of lncRNAs ($LS$) and miRNAs ($MS$) are measured by pairwise comparisons of sequences using the Levenshtein distance, as detailed in Supplementary file S1(A). The expression profile similarities of lncRNAs ($LE$) and miRNAs ($ME$) are computed based on the Pearson correlation coefficient between their expression profiles, with further details provided in Supplementary file S1(B). Lastly, the GIP kernel similarities of lncRNAs ($LG$) and miRNAs ($MG$) are calculated by Gaussian kernel function analysis of the lncRNA–miRNA interaction network, as described in Supplementary file S1(C).

#### Feature extraction from the multimodal similarity matrices

To mitigate the influence of low-similarity associations, we adopt a p-nearest neighbor graph approach [28] to refine the original similarity matrices before input into the GCN. This strategy preserves highly similar local neighbors, thereby maintaining proximity among closely related samples within the feature spaces and enhancing the preservation of local geometric structures [29, 30]. As a result, refined affinity graphs—$LS^{*}$, $LE^{*}$, and $LG^{*}$ for lncRNAs and $MS^{*}$, $ME^{*}$, and $MG^{*}$ for miRNAs—are constructed. Further details on the p-nearest neighbor graph method are provided in Supplementary file S1(D).

Next, based on these affinity graphs, GCN is adopted to learn the feature representation of the nodes. Taking $LS^{*}$ as an example, the feature representations are obtained through Equation (1).


(1) \begin{align*}& L_{LS^{*}}^{(l+1)} = \sigma \left(D_{LS^{*}}^{-\frac{1}{2}}S_{LS^{*}}^{^{\prime}}D_{LS^{*}}^{-\frac{1}{2}}L_{LS^{*}}^{(l)}W_{LS^{*}}^{(l)}\right)\end{align*}

Here, $L_{LS^{*}}^{(l)}$ represents the embedding of lncRNA obtained by the $l$th layer of GCN on $LS^{*}$. $L_{LS^{*}}^{(0)}$ is the initial embedding of lncRNA. $S_{LS^{*}}^{^{\prime}} = I + S_{LS^{*}}$ where $I$ represents the identity matrix, $S$ is the adjacency matrix of the affinity graphs ( $LS^{*}$, $LE^{*}$, $LG^{*}$ for lncRNAs, $MS^{*}$, $ME^{*}$, $MG^{*}$ for miRNAs), and $D_{LS^{*}}$ denotes the diagonal matrix. The affinity graphs $LS^{*}$, $LE^{*}$, and $LG^{*}$ processed by GCN are denoted as $L_{LS^{*}}^{GCN}$, $L_{LE^{*}}^{GCN}$, and $L_{LG^{*}}^{GCN}$, respectively. The feature vector for lncRNAs can be represented as ${L_{GCN}} = \{ L_{L{S^{*}}}^{GCN},L_{L{E^{*}}}^{GCN},L_{L{G^{*}}}^{GCN}\}$. Similarly, the feature tensor of miRNA is marked as ${M_{GCN}} = \{ M_{M{S^{*}}}^{GCN}, M_{M{E^{*}}}^{GCN}, M_{M{G^{*}}}^{GCN}\}$.

### Long-distance dependency features

#### One-hot encoding of sequences with different scale k-mers

To capture sequence features at the multiscale $\text{k}$-mer level, as mentioned in literature [24], the sequences of miRNAs and lncRNAs are first aligned in length by duplicating and truncating them. Next, the aligned sequences are cut into different scale $\text{k}$-mers ($\text{k}$=2,3,4) segments, and represented by positional vectors of k-mers through one-hot encoding. Then, the positional vectors of $\text{k}$-mers at scales 2,3, and 4 are mapped to unified-dimensional feature vectors via an embedding layer. Based on the unified positional vectors, encoding matrices of lncRNAs and miRNAs are obtained and represented as $L_{k2}, L_{k3}, L_{k4}$ for lncRNA and $M_{k2}, M_{k3}, M_{k4}$ for miRNA.

#### Feature extraction from multiscale encoding matrices

As described in Equation (2) and Equation (3), the positional embeddings of $\text{k}$-mers are initially extracted from the encoding matrices utilizing the sine and cosine functions, respectively.


(2) \begin{align*} & {PE_{pos,2a} = \sin \left( {\frac{{pos}}{{{{10000}^{2a/dmodel}}}}} \right)} \end{align*}

(3) \begin{align*} & {PE_{pos,2a+1} = \cos \left( {\frac{{pos}}{{{{10000}^{2a/dmodel}}}}} \right)} \end{align*}

In Equation (2) and Equation (3), $pos$ represents the position index of the $\text{k}$-mer in the sequence, $a$ represents the dimension index, and $dmodel$ represents the embedding dimension. After that, the encoding matrices at different $\text{k}$-mer scales 2,3 and 4 for lncRNAs are marked as $L_{k2^{*}}\!, L_{k3^{*}}\!, L_{k4^{*}}\!$. Similarly, the encoding matrices for miRNAs are represented as $M_{k2^{*}\!}, M_{k3^{*}\!}, M_{k4^{*}\!}$.

Next, encoding matrices of lncRNAs and miRNAs are fed into the Transformer to learn their long-distance dependency features. In the transformer, the encoder consists of a stack of $n$ identical layers, including a multihead attention module and a feed-forward network. Taking the encoding matrix $L_{k2^{*}}$ as an example, it can be updated by the multi-head attention mechanism represented by Equation (4), where $head_{i}$ is computed using Equation (5). In Equation (5), $W_{i}^{Q}$, $W_{i}^{K}$ and $W_{i}^{V}$ are learnable parameters, $1 \le i \le{n_{head}}$, ${n_{head}}$ is refers to the number of attention heads, and $d_{K}$ is the feature dimension of K. Finally, as shown by Equation (6), the embeddings are generated by residual connection and layer normalization. Then, the feature representation $L_{k2^{*}}^{trans}$ of the encoding matrix $L_{k2^{*}}$ is obtained through a two-layer feed-forward network which is described as Equation (7).


(4) \begin{align*} & {\text{MultiHeadAttention}}({L_{k{2^{*}}}}) = {\text{concat}}\left(hea{d_{1}}, \cdot \cdot \cdot,hea{d_{{n_{head}}}}\right){W^{O}} \end{align*}

(5) \begin{align*} & hea{d_{i}} = {\text{softmax}}\left( {\frac{{{L_{k{2^{*}}}}W_{i}^{Q}{{\left( {{L_{k{2^{*}}}}W_{i}^{K}} \right)}^{T}}}}{{\sqrt{{d_{{K_{i}}}}}}}} \right){L_{k{2^{*}}}}W_{i}^{V} \end{align*}

(6) \begin{align*} & L_{k2^{*}}^{^{\prime}} = {\text{layerNorm}}\left( {{L_{k{2^{*}}}} + {\text{MultiHeadAttention}}({L_{k{2^{*}}}})} \right) \end{align*}

(7) \begin{align*} & L_{k{2^{*}}}^{trans} = {\text{layerNorm}}\left( {L_{k2^{*}}^{^{\prime}} + {\text{Relu}}\left( {\,L_{k{2^{*}}}^{^{\prime}}{W_{1}} + {b_{1}}} \right){W_{2}} + {b_{2}}} \right) \end{align*}

Likewise, $L_{k3^{*}}^{trans}$ and $L_{k4^{*}}^{trans}$ can also be learned from $L_{k3^{*}}$ and $L_{k4^{*}}$ by the Transformer, respectively. The long-distance dependency features of lncRNA can be reprensented by ${L_{trans}} = \{ L_{k2^{*}}^{trans},L_{k3^{*}}^{trans},L_{k4^{*}}^{trans}\}$. As for miRNAs, their long-distance dependency features can be denoted by ${M_{trans}} = \{ M_{k2^{*}}^{trans}, M_{k3^{*}}^{trans}, M_{k4^{*}}^{trans}\}$.

### Multichannel attention mechanism

The multichannel attention mechanism [31] is adopted to capture the representative features of lncRNAs and miRNAs adaptively from $L_{GCN}$, $M_{GCN}$, $L_{trans}$, and $M_{trans}$. To simplify the description, the above feature matrices are marked as $X\in \mathbb{R}^{t\times m\times n}$ when they are processed by the multichannel attention mechanism, where $t$ represents the number of feature submatrices and $m\times n$ is the dimensions of the submatrices. Each feature submatrix (eg. $L_{k2^{*}}^{trans},L_{k3^{*}}^{trans}$ or $L_{k4^{*}}^{trans}$ of $L_{trans}$) is regarded as an independent channel and compressed to measure the channel attention. The $c$th feature submatrix is marked as $x_{c}\in \mathbb{R}^{m\times n}$, and its corresponding channel representation $z_{c}$ can be extracted using Equation (8). The attention weight $z_{c}^{att}$ for the channel is calculated using Equation (9), where $\delta $ is the sigmoid activation function, $\sigma $ represents the ReLU activation function, and $W_{1}$, $W_{2}$ are the training parameters.


(8) \begin{align*} & {{z_{c}} = {\varphi_{sq}}\left( {x_{c}} \right) = \frac{1}{{{m} \times n}}\sum\nolimits_{i = 1}^{{m}} {\sum\nolimits_{j = 1}^{n} {x_{c}\left( {i,j} \right)}} } \end{align*}

(9) \begin{align*} & {z_{c}^{att} = {\varphi_{att}}\left( {{z_{c}},W} \right) = {{\delta}}\left( {{W_{2}}\sigma \left( {{W_{1}}{z_{c}}} \right)} \right)} \end{align*}

Then, to integrate features from all channels, a convolution operation is performed using Equation (10) where $x_{c}^{*}$ is measured by Equation (11).


(10) \begin{align*} & X^{*} = {\text{conv}}\left({x_{1}^{*},x_{2}^{*},\ldots,x_{t}^{*}}\right) \end{align*}

(11) \begin{align*} & {x_{c}^{*} = {\varphi_{scale}}\left( {x_{c},z_{c}^{att}} \right) = z_{c}^{att} \cdot x_{c}} \end{align*}

Finally, the representative features of lncRNAs and miRNAs are obtained and denoted as $L_{GCN}^{*}$, $M_{GCN}^{*}$, $L_{trans}^{*}$, and $M_{trans}^{*}$, respectively.

### Multimodal contrastive representations learning

**Figure 2:**
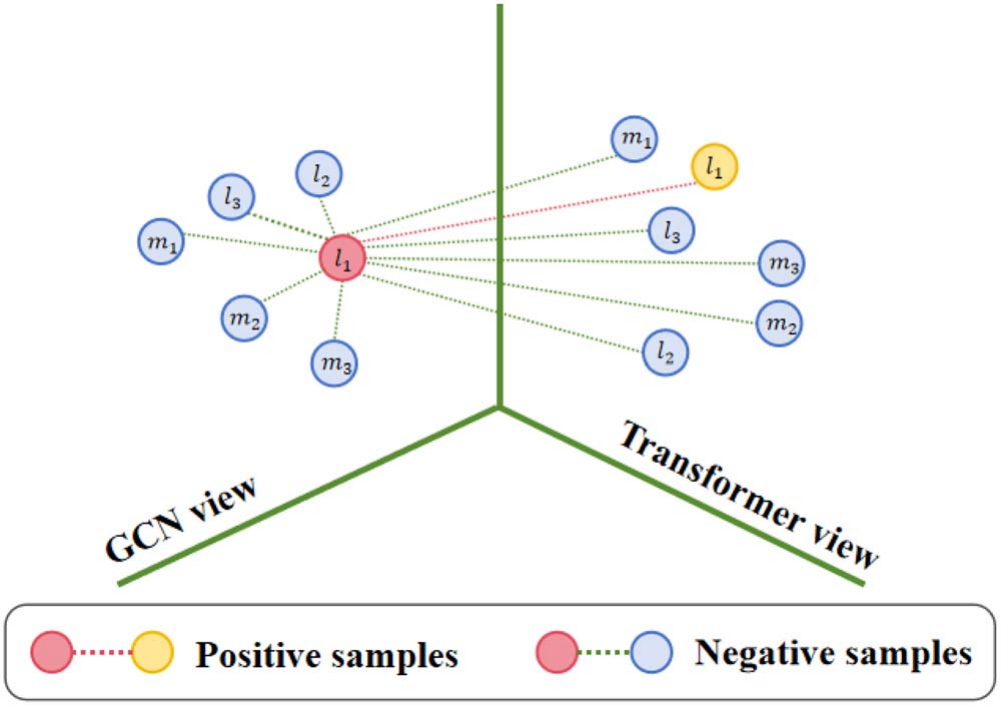
The relationship of the anchor and its positive and negative samples.

To obtain the discriminative features, a contrastive learning method [32] is exploited to effectively integrate local neighborhood structural features and long-distance dependency features of lncRNAs and miRNAs. Figure 2 illustrates the relationship of the anchor and its positive and negative samples. Positive samples contain the specific anchor (lncRNA/miRNA) from the GCN view (local neighborhood structural features) and the Transformer view (long-distance dependency features). Negative samples consist of the anchor and the other lncRNA/miRNA. For example, $l_{1}$ in the GCN view is selected as the anchor. $l_{1}$ in the Transformer view, paired with the anchor, forms a positive sample. The anchor paired with $l_{2}$, $l_{3}$, $m_{1}$, etc., are treated as negative samples.

The distance between positive and negative samples is defined by the cosine similarity of their feature vectors. Equation (12) represents the loss function of this method, where $T$ denotes the temperature coefficient. For each anchor $r$, $z_{r}$ represents the feature vector of anchor $r$, $P(r)$ refers to the set of positive samples, $|P(r)|$ is the number of positive samples, $z_{p}$ represents the feature vector of a positive sample paired with the anchor, $N(r)$ is the set of negative samples, $z_{n}$ represents the feature vector of a negative sample paired with the anchor.


(12) \begin{align*} & {L_{con}} = \sum\nolimits_{r \in R} {\frac{{ - 1}}{{\left| {P\left( r \right)} \right|}}} \sum\nolimits_{p \in P\left( r \right)} {\log \frac{{\exp \left( {{z_r} \cdot{{{z_p}} \mathord{\left/ {\vphantom{{{z_p}} T}} \right. \!} T}} \right)}}{{\sum\nolimits_{n \in \left( {N\left( r \right) \cup P\left( r \right)} \right)} {\exp \left( {{z_r} \cdot{{{z_n}} \mathord{\left/ {\vphantom{{{z_n}} T}} \right. \!} T}} \right)} }}} \end{align*}

### LMI prediction

In contrast to MLPs, KANs utilize adaptive learnable activation functions on edges, whereas MLPs use fixed activation functions on nodes [33]. Therefore, KANs may eliminate the influence of vector concatenation order, facilitating more effective integration of lncRNA and miRNA feature representations for a more accurate characterization of LMI. In our study, $L_{GCN}^{*}$ and $L_{trans}^{*}$ are concatenated to obtain $L^{*}$, and $M_{GCN}^{*}$ and $M_{trans}^{*}$ are concatenated to obtain $M^{*}$. The KAN layer can be expressed by Equation (13), where $ \sigma $ denotes the SiLU activation function, $B(x)$ represents the B-spline function, and $W_{base}$ and $W_{spline}$ refer to the base weights and spline weights, respectively. To predict potential LMIs, the scores $\hat y$ of candidate LMIs are calculated by Equation (14), where $ \sigma $ represents the sigmoid activation function.


(13) \begin{align*} & {\text{KAN(x)}} = W_{base} \cdot{{\sigma}}(x) + W_{spline} \cdot B(x) \end{align*}

(14) \begin{align*} & {\hat y= {{\sigma }}({\text{KAN}}({\text{concat}}({L^{*}},{M^{*}})))} \end{align*}

In this study, binary cross-entropy (BCE) loss is utilized as the objective function and minimized through Equation (15). In Equation (15), $N$ denotes the number of samples, $y \in \left \{ 0,1 \right \}$ represents the true label, $\hat{y}_{i}$ is the predicted label.


(15) \begin{align*}& {{L_{BCE}} = - \frac{1}{{{N}}}\sum\limits_{i}^{N} {{y_{i}}\log \left( {\mathop{{y_{i}}}\limits^ \wedge } \right)} + \left( {1 - {y_{i}}} \right)\log \left( {1 - \mathop{{y_{i}}}\limits^ \wedge } \right)}\end{align*}

Accordingly, the total loss function is defined as Equation (16), and a novel LMIs predictor is trained. If $\hat{y}_{i}$ exceeds 0.5, it can be inferred that lncRNA and miRNA interact with each other. Otherwise, it is considered that no interaction exists.


(16) \begin{align*}& {L_{total}} = {L_{con}} + {L_{BCE}}\end{align*}

## Experimental results and analysis

### Datasets and evaluation metrics

This study uses the data introduced in previous literature [17,34]. LncRNA sequences are downloaded from the GENCODE [35] and LNCipedia [36] database, and miRNA sequences are downloaded from the miRbase [37] database. LncRNA expression profiles are downloaded from the NONCODE v5 [38] database, and the miRNA expression profiles are obtained from the lncRNASNP2 [39] database. After redundancy removal and data alignment, 270 miRNAs and 1,643 lncRNAs remain. To construct a negative sample dataset, lncRNAs and miRNAs were randomly paired to form lncRNA-miRNA pairs, among which the ones absent in the positive sample set are considered negative samples. Then, three datasets namely DS-R1, DS-R5, and DS-R10, are constructed with positive-to-negative sample ratios of 1:1, 1:5, and 1:10 for experiments, respectively. Specifically, DS-R1 includes 7,500 positive and 7,500 negative samples, DS-R5 contains 1,500 positive and 7,500 negative samples, and DS-R10 has 750 positive and 7,500 negative samples.

In this study, the performance of the models is evaluated using metrics including accuracy (ACC), recall, precision, f1-score, area under the receiver operating characteristic (ROC) curve (AUC), and area under the precision-recall (PR) curve (AUPR).

### Comparison with the concerned methods

**Figure 3:**
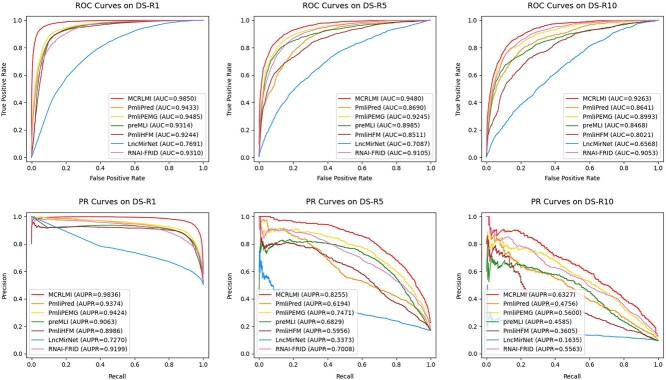
The ROC and PR curves of the concerned methods in predicting LMIs on benchmark datasets.

To assess the effectiveness of our model, MCRLMI is compared with six existing LMI prediction methods, including PmliPred [19], PmliPEMG [21], preMLI [11], PmliHFM [24], LncMirNet [17], and RNAI-FRID [22]. The performance of the methods in predicting LMIs is estimated on benchmark datasets DS-R1, DS-R5, and DS-R10 by five-fold cross-validation. Table 1 presents the average performance and deviation of the methods for each metric, with the best results highlighted in bold and the second-best results underlined. Figure 3 shows the corresponding ROC and PR curves for the concerned methods.

**Table 1:** The performance of the concerned methods in predicting LMIs under the five-fold cross-validation

Datasets	Model	ACC	recall	precision	f1-score
	PmliPred	0.8770$\pm $0.0069	0.8985$\pm $0.0065	0.8615$\pm $0.0095	0.8796$\pm $0.0064
	PmliPEMG	0.8935$\pm $0.0034	0.9059$\pm $0.0080	0.8840$\pm $0.0080	0.8948$\pm $0.0033
	preMLI	0.8779$\pm $0.0063	0.8776$\pm $0.0091	0.8781$\pm $0.0063	0.8778$\pm $0.0065
DS-R1	PmliHFM	0.8733$\pm $0.0071	0.9121$\pm $0.0081	0.8464$\pm $0.0064	0.8781$\pm $0.0070
	LncMirNet	0.6965$\pm $0.0034	0.8263$\pm $0.0073	0.6560$\pm $0.0022	0.7313$\pm $0.0038
	RNAI-FRID	0.8566$\pm $0.0032	0.8728$\pm $0.0070	0.8455$\pm $0.0056	0.8589$\pm $0.0032
	MCRLMI	**0.9417$\pm $0.0039**	**0.9455$\pm $0.0057**	**0.9383$\pm $0.0041**	**0.9419$\pm $0.0039**
	PmliPred	0.8696$\pm $0.0018	0.3967$\pm $0.0122	0.6891$\pm $0.0140	0.5033$\pm $0.0088
	PmliPEMG	0.9050$\pm $0.0030	**0.6460$\pm $0.0086**	0.7497$\pm $0.0153	**0.6939$\pm $0.0078**
	preMLI	0.8963$\pm $0.0024	0.6420$\pm $0.0201	0.7087$\pm $0.0081	0.6735$\pm $0.0112
DS-R5	PmliHFM	0.8624$\pm $0.0014	0.2420$\pm $0.0069	0.7830$\pm $0.0214	0.3696$\pm $0.0077
	LncMirNet	0.7786$\pm $0.0060	0.3993$\pm $0.0076	0.3544$\pm $0.0124	0.3755$\pm $0.0098
	RNAI-FRID	0.8844$\pm $0.0024	0.3967$\pm $0.0062	0.8163$\pm $0.0304	0.5336$\pm $0.0039
	MCRLMI	**0.9124$\pm $0.0028**	0.5567$\pm $0.0160	**0.8716$\pm $0.0093**	0.6793$\pm $0.0129
	PmliPred	0.9178$\pm $0.0009	0.1747$\pm $0.0145	0.6892$\pm $0.0094	0.2785$\pm $0.0190
	PmliPEMG	0.9285$\pm $0.0013	0.3467$\pm $0.0170	0.7227$\pm $0.0172	0.4683$\pm $0.0153
	preMLI	0.9168$\pm $0.0012	**0.4653$\pm $0.0145**	0.5507$\pm $0.0092	**0.5043$\pm $0.0078**
DS-R10	PmliHFM	0.9142$\pm $0.0005	0.0853$\pm $0.0073	0.7444$\pm $0.0193	0.1530$\pm $0.0119
	LncMirNet	0.7936$\pm $0.0049	0.3360$\pm $0.0192	0.1729$\pm $0.0049	0.2283$\pm $0.0080
	RNAI-FRID	0.9208$\pm $0.0005	0.1733$\pm $0.0082	**0.7981$\pm $0.0169**	0.2847$\pm $0.0107
	MCRLMI	**0.9314$\pm $0.0016**	0.3547$\pm $0.0152	0.7643$\pm $0.0157	0.4844$\pm $0.0162

As shown in Table 1, MCRLMI achieved ACC of 0.9417, recall of 0.9455, precision of 0.9383, and f1-score of 0.9419 on the DS-R1 dataset, improving by 4.82%, 3.34%, 5.43%, and 4.71% compared to the second-best performing method, respectively. Similarly, as seen in Fig. 3, MCRLMI achieved AUC of 0.9850 and AUPR of 0.9836 on DS-R1, demonstrating a significant improvement over other methods. These results indicate that MCRLMI performs significantly better on the balanced dataset.

However, as the proportion of negative samples increases, all compared models show varying degrees of performance degradation on the DS-R5 and DS-R10 datasets, particularly in metrics sensitive to the minority class, such as recall, precision, F1-score, and AUPR. This trend is primarily due to the bias induced by class imbalance during training, which leads the model to excessively focus on learning negative class characteristics, thereby increasing the number of false negatives and false positives and severely affecting the recognition of positive samples. In contrast, the accuracy is less affected by the class imbalance and even shows a slight improvement. This is because the dominance of negative samples increases the proportion of true negatives, which, in turn, masks the decrease in the model’s ability to identify positive class samples. And MCRLMI still outperforms other methods in the two imbalanced datasets, in general. In summary, class imbalance weakens the ability of all models to recognize positive samples, thus affecting overall classification performance.

### Ablation study

#### The impact of feature learning strategies

To assess the impact of feature learning strategies, six variants of MCRLMI are evaluated on DS-R1 using five-fold cross-validation. w/o GA indicates that the multichannel attention mechanism is not applied to the GCN output. Instead, the three feature representations are fused using a simple summation. w/o TA means the multichannel attention mechanism is not applied to the Transformer output, and the three feature representations are fused through summation. These two variants are designed to evaluate the impact of the multichannel attention mechanism. w/o Contrast means contrastive learning is removed from MCRLMI. w/o p-nearest indicates that p-nearest is removed in MCRLMI. w/o GCN means MCRLMI relies solely on the Transformer to capture long-distance dependency features, while w/o Trans denotes it depends only on the GCN to capture local neighborhood structural features. The last two variants of MCRLMI focus on extracting features from a single perspective.

**Figure 4:**
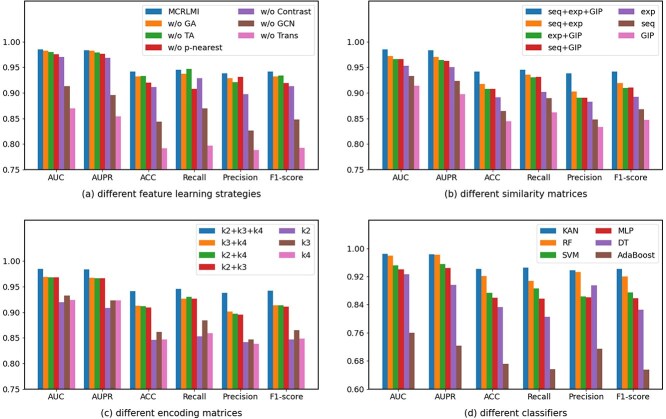
The results of ablation studies for MCRLMI.

The experimental results of MCRLMI and its variants are shown in Fig. 4(a). It can be observed that MCRLMI outperforms the other variants. Among the variants, w/o Trans and w/o GCN are inferior to others, with AUC dropping by 11.49% and 7.2%. This highlights the importance of capturing features from multiple perspectives. The performance of w/o GA and w/o TA suggests that multichannel attention provides a slight boost, while w/o Contrast indicates that contrastive learning enhances feature representation. Overall, every feature learning strategy in MCRLMI is crucial to its effectiveness.

#### The impact of similarity matrices

To explore the impact of different similarity matrices, MCRLMI is evaluated on DS-R1 using five-fold cross-validation, and the results are shown in Fig. 4(b). In Fig. 4(b), exp represents expression profile similarity, seq denotes sequence similarity, and GIP refers to GIP kernel similarity. MCRLMI using expression profile similarity performs best with a single similarity, indicating that it captures richer biological information by reflecting gene expression across conditions. Combining two similarities further enhances feature learning but still falls slightly short of MCRLMI with all three similarities. Overall, incorporating all three similarities provides more comprehensive lncRNA and miRNA representations, improving prediction accuracy.

#### The impact of encoding matrices

To investigate the contribution of different encoding matrices, we compare MCRLMI with various encoding matrices on DS-R1 using five-fold cross-validation, and the results are presented in Fig. 4(c). k2, k3, and k4 represent the encoding matrices derived from $\text{k}$-mer of sequences when $\text{k}$=2, 3, and 4, respectively. The results show that MCRLMI based on k3 achieves the highest AUC value of 0.9326 when using a single encoding matrix. When using pairs of encoding matrices, the combination of k3 and k4 (k3+k4) achieves the best performance, though the AUPR values across all pairs differ by no more than 0.13%. Notably, all pairwise combinations outperform the use of a single encoding matrix. However, the highest performance is achieved when all three encoding matrices (k2+k3+k4) are incorporated into MCRLMI.

#### The impact of classifiers

To evaluate the impact of different classifiers on model performance, we conducted five-fold cross-validation on the DS-R1 dataset, comparing six classifiers: KAN, SVM, Random Forest (RF), Decision Tree (DT), AdaBoost, and Multi-Layer Perceptron (MLP). As shown in Fig. 4(d), KAN outperformed all other methods across all evaluation metrics. Compared with the second-best classifier, KAN achieved improvements of 0.55%, 0.23%, 2.09%, 3.78%, 0.61%, and 2.22% in AUC, AUPR, ACC, recall, precision, and f1-score, respectively. This superior performance is primarily attributed to KAN’s powerful nonlinear modeling capability and interpretability. Based on the Kolmogorov–Arnold representation theorem, KAN decomposes multivariate functions into compositions of univariate functions by leveraging learnable spline-based activation functions, without relying on conventional linear weight combinations. This architectural design makes KAN particularly well suited for complex feature representation and high-dimensional nonlinear mapping tasks [33].

#### The summary of ablation study results

Each component in MCRLMI contributes to the overall performance to varying degrees. Notably, the combined use of GCN and Transformer plays a pivotal role in model performance, as removing either component results in a significant decline in AUC (by 11.49% and 7.2%, respectively). This highlights the importance of multi-perspective feature extraction in enhancing the accuracy of lncRNA–miRNA interaction predictions. In comparison, contrastive learning and multi-channel attention contribute relatively less to the overall performance. Moreover, multi-source heterogeneous data fusion proves to be an effective strategy. Among the similarity matrices, expression profile similarity achieves the best performance when used individually. Within the encoding matrices, the k-mer encoding with k=3 produces the most optimal results in single-feature scenarios.

### Parameter sensitivity analysis

**Figure 5:**
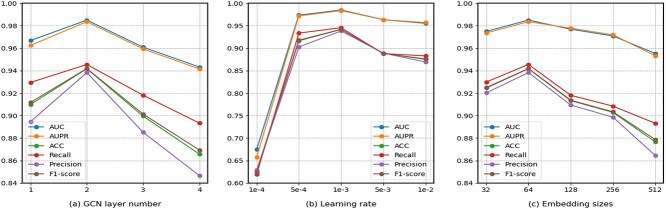
The parameter sensitivity analysis with different numbers of convolution layers, learning rates, and embedding sizes.

In this section, we perform a parameter sensitivity analysis to examine the impact of the number of graph convolution layers, learning rate, and embedding size. Firstly, we evaluate the performance of MCRLMI using 1, 2, 3, and 4 graph convolution layers, respectively. As shown in Fig. 5(a), MCRLMI achieves the best performance with two convolution layers, but the performance declines when more layers are added. This decline may be attributed to an increasing amount of irrelevant neighborhood information being incorporated into the target node as more layers are stacked [40]. Secondly, we evaluate the performance of MCRLMI with different learning rates, selecting from 0.0001, 0.0005, 0.001, 0.005, and 0.01. The results of the experiment are shown in Fig. 5(b). The performance increases significantly from 1e-4 to 5e-4, achieving the best results at 1e-3, before gradually declining from 1e-3 to 1e-2. Finally, we investigate the impact of embedding size, selecting from 32, 64, 128, 256, and 512. The experimental results in Fig. 5(c) show that MCRLMI performs best with an embedding size of 64.

### Performance of MCRLMI under cold start

In LMI research, the cold-start problem refers to the challenge of predicting potential associations for newly identified lncRNAs (or miRNAs) due to the absence of their known interactions. Specifically, this problem involves handling novel lncRNAs (or miRNAs) that are not present in the training dataset and accurately predicting their interactions with miRNAs (or lncRNAs). In this section, we assessed MCRLMI and the methods discussed in Section 3.2 on DS-R1 using five-fold cross-validation under cold-start experiments to investigate their generalization capability. As literature [41], in the lncRNA-centric cold-start experiment, lncRNAs present in the training set are ensured to have no associated interactions in the validation set. Similarly, in the miRNA-centric cold-start experiment, miRNAs included in the training set must not appear in any interactions in the validation set. In hot-start experiments, the above constraints are not applied. These experiments are designed to simulate real-world scenarios for discovering novel LMIs.

The experimental results are presented in Table 2, with the best results highlighted in bold and the second-best results underlined. It can be observed that MCRLMI achieves the best overall performance across all three experimental settings. For almost every metric, all methods perform better under hot-start than under cold-start. This is because some lncRNAs or miRNAs in the validation set may also appear in the training set (excluding interaction pairs) in the hot-start scenario, allowing MCRLMI to leverage learned features and previous interaction patterns for better prediction. In contrast, the cold-start scenario involves entirely unseen molecules, lacking historical context or prior knowledge, which significantly increases prediction difficulty.

**Table 2:** Performance comparison of the concerned LMI predictors under hot-start and cold-start

Setting	Model	AUC	AUPR	ACC	recall	precision	f1-score
	PmliPred	0.9452	0.9395	0.8770	0.8985	0.8622	0.8794
	PmliPEMG	0.9496	0.9438	0.8935	0.9059	0.8844	0.8947
	preMLI	0.9320	0.9070	0.8779	0.8776	0.8782	0.8778
Hot-start	PmliHFM	0.9244	0.8986	0.8733	0.9121	0.8464	0.8781
	LncMirNet	0.7740	0.7329	0.6965	0.8263	0.6568	0.7312
	RNAI-FRID	0.9310	0.9202	0.8566	0.8728	0.8455	0.8589
	MCRLMI	**0.9850**	**0.9836**	**0.9417**	**0.9455**	**0.9383**	**0.9419**
	PmliPred	0.9140	0.9043	0.7432	0.5751	**0.8951**	0.6412
	PmliPEMG	0.9250	0.9158	0.8560	0.8708	0.8472	0.8583
	preMLI	0.8759	0.8514	0.7927	0.8851	0.7482	0.8105
lncRNA-centric cold-start	PmliHFM	0.6182	0.5707	0.6142	**0.9318**	0.5777	0.7087
	LncMirNet	0.7635	0.7351	0.6910	0.8145	0.6534	0.7250
	RNAI-FRID	0.9069	0.8910	0.8325	0.8699	0.8100	0.8387
	MCRLMI	**0.9592**	**0.9574**	**0.8913**	0.9247	0.8668	**0.8947**
	PmliPred	0.9165	0.9130	0.7572	0.5982	**0.9020**	0.6839
	PmliPEMG	0.9282	0.9224	0.8597	0.8490	0.8658	0.8563
	preMLI	0.8786	0.8629	0.8021	0.8366	0.7826	0.8085
miRNA-centric cold-start	PmliHFM	0.8375	0.7702	0.8273	0.8999	0.8007	0.8467
	LncMirNet	0.7583	0.7334	0.6845	0.8085	0.6484	0.7185
	RNAI-FRID	0.9107	0.8994	0.8350	0.8434	0.8302	0.8364
	MCRLMI	**0.9569**	**0.9571**	**0.8901**	**0.9207**	0.8677	**0.8934**

### Visualization

**Figure 6:**
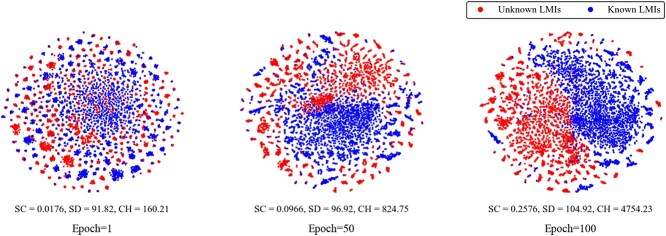
Visualization of the learned lncRNA-miRNA embeddings by MCRLMI under different epochs.

To show the performance of the model more intuitively, we carry out visualization experiments to show the embedding learning process of candidate lncRNA-miRNA interactions. Specifically, positive samples were marked as blue points and negative samples were marked as red points. Their embeddings were visualized at different stages using the t-SNE tool [42]. The results shown in Fig. 6 illustrate that the distribution of positive and negative samples is unorganized at epoch=1. As the number of epochs increases, the positive and negative samples gradually begin to cluster. By epoch=100, they are almost completely clustered into separate regions. However, it is noticeable that red and blue points still overlap in some regions, suggesting that the decision boundary for LMIs remains challenging. This observation further confirms that the learned embeddings of lncRNA-miRNA pairs are discriminative and interpretable, ultimately enhancing the performance of MCRLMI in predicting LMIs.

In addition, to assess the clustering quality and class separation of positive and negative samples in the embedding space, we introduced three quantitative metrics: the Silhouette Coefficient (SC) [43], the Separation Distance (SD) and Calinski-Harabasz (CH) Index [44]. As shown in Fig. 6, both SC, SD, and CH values increase as training progresses, suggesting that MCRLMI progressively optimizes the embedding representations and enhances its ability to distinguish.

### Case study

In this section, we conduct case studies on DS-R1 to further demonstrate the practicality of MCRLMI. In our studies, lncRNA NONHSAT137542.2 (XIST) and miRNA hsa-miR-590-3p are selected as the targets because of their significant roles in regulating human diseases. For example, as a tumor promoter in non-small cell lung cancer (NSCLC), XIST is overexpressed, promoting the proliferation and migration of NSCLC cells [45]. The hsa-miR-590-3p has been shown to promote the proliferation, migration, and invasion of pancreatic cancer cells [46] and serves as a sensitive biomarker for patients with colorectal cancer [47]. In our experiments, we first remove interactions involving the specified target molecules from DS-R1 and train MCRLMI using the remaining data. The trained model is then used to predict miRNAs and lncRNAs that interact with the given targets. Next, the predicted lncRNAs/miRNAs are ranked in descending order based on their prediction scores. Finally, the prediction accuracy is assessed by verifying whether the predicted interactions are documented in existing databases. The top 20 predicted miRNAs associated with NONHSAT137542.2 are presented in Table 3, of which 17 have been validated in the lncRNASNP2 database. Table 4 illustrates the top 20 predicted lncRNAs associated with miRNA hsa-miR-590-3p, of which 14 have been validated in the lncRNASNP2 database.

**Table 3:** The top 20 miRNAs predicted by MCRLMI that are associated with lncRNA NONHSAT137542.2

Rank	miRNA	Evidence	Rank	miRNA	Evidence
1	hsa-miR-1297	unknown	11	hsa-miR-455-5p	unknown
2	hsa-miR-381-3p	lncRNASNP2	12	hsa-miR-26a-5p	unknown
3	hsa-miR-136-5p	lncRNASNP2	13	hsa-miR-211-5p	lncRNASNP2
4	hsa-miR-125b-5p	lncRNASNP2	14	hsa-miR-29b-3p	lncRNASNP2
5	hsa-miR-876-5p	lncRNASNP2	15	hsa-miR-338-3p	lncRNASNP2
6	hsa-miR-367-3p	lncRNASNP2	16	hsa-miR-363-3p	lncRNASNP2
7	hsa-miR-135a-5p	lncRNASNP2	17	hsa-miR-329-3p	lncRNASNP2
8	hsa-miR-29a-3p	lncRNASNP2	18	hsa-miR-196a-5p	lncRNASNP2
9	hsa-miR-129-5p	lncRNASNP2	19	hsa-miR-490-3p	lncRNASNP2
10	hsa-miR-485-5p	lncRNASNP2	20	hsa-miR-486-5p	lncRNASNP2

**Table 4:** The top 20 lncRNAs predicted by MCRLMI that are associated with miRNA hsa-miR-590-3p

Rank	lncRNA	Evidence	Rank	lncRNA	Evidence
1	NONHSAT130414.2	unknown	11	NONHSAT007662.2	lncRNASNP2
2	NONHSAT007698.2	lncRNASNP2	12	NONHSAT007667.2	lncRNASNP2
3	NONHSAT094688.2	unknown	13	NONHSAT113469.2	unknown
4	NONHSAT007681.2	lncRNASNP2	14	NONHSAT007695.2	lncRNASNP2
5	NONHSAT007671.2	lncRNASNP2	15	NONHSAT114486.2	unknown
6	NONHSAT007664.2	lncRNASNP2	16	NONHSAT007693.2	lncRNASNP2
7	NONHSAT007701.2	lncRNASNP2	17	NONHSAT007672.2	lncRNASNP2
8	NONHSAT007670.2	lncRNASNP2	18	NONHSAT007673.2	lncRNASNP2
9	NONHSAT145164.2	unknown	19	NONHSAT017462.2	unknown
10	NONHSAT007688.2	lncRNASNP2	20	NONHSAT007684.2	lncRNASNP2

Based on literature data and functional annotations of GO, we further analyzed interactions with ’unknown’. From the available information, we found that hsa-miR-26a-5p, as shown in Table 3, shares the GO term GO:0060255 with NONHSAT137542.2. This term is related to the regulation of macromolecule metabolic processes, including proteins, nucleic acids, and polysaccharides. This suggests that although some interactions have not been experimentally confirmed, the functional similarity indicates their potential biological relevance. It further demonstrates the utility of the model in discovering potential functional associations.

Moreover, we also analyzed the interactions predicted as ‘unknown’ on the DS-R5 and DS-R10. The results and corresponding discussion are included in Supplementary File S1(E).

### Time complexity analysis

In this section, we analyze the time complexity of the MCRLMI. As shown in Fig. 1, MCRLMI consists of four main modules: a GCN to extract local neighborhood structural features, a Transformer to capture long-distance dependency features, a multi-channel attention mechanism for feature fusion, and contrastive learning followed by the final prediction. Assuming the number of lncRNAs is $N$, the number of miRNAs is $M$, the number of samples is $S$, and the length of sequences is $L$.

For GCN, the complexity is $\mathcal{O}(\left |E_{m}\right |+\left |E_{l}\right |)$, where $\left |E_{l}\right |$ and $\left |E_{m}\right |$ denote the number of edges in the lncRNA and miRNA graphs, respectively. For Transformer, the complexity of the multi-head attention mechanism is $\mathcal{O}(S \cdot L^{2})$, and the feed-forward network is $\mathcal{O}(S \cdot L)$. Thus, the overall complexity of the Transformer is $\mathcal{O}(S \cdot L^{2})$. The complexity of the multi-channel attention mechanism is $\mathcal{O}(3\cdot (M+N))$. During the contrastive learning stage, the complexity is $\mathcal{O}(N^{2}+M^{2})$. In the prediction stage, the complexity is dominated by the KAN layer, given by $\mathcal{O}(S)$. To sum up, the complexity of the MCRLMI can be expressed as $\mathcal{O}(S\cdot L^{2})$.

**Figure 7:**
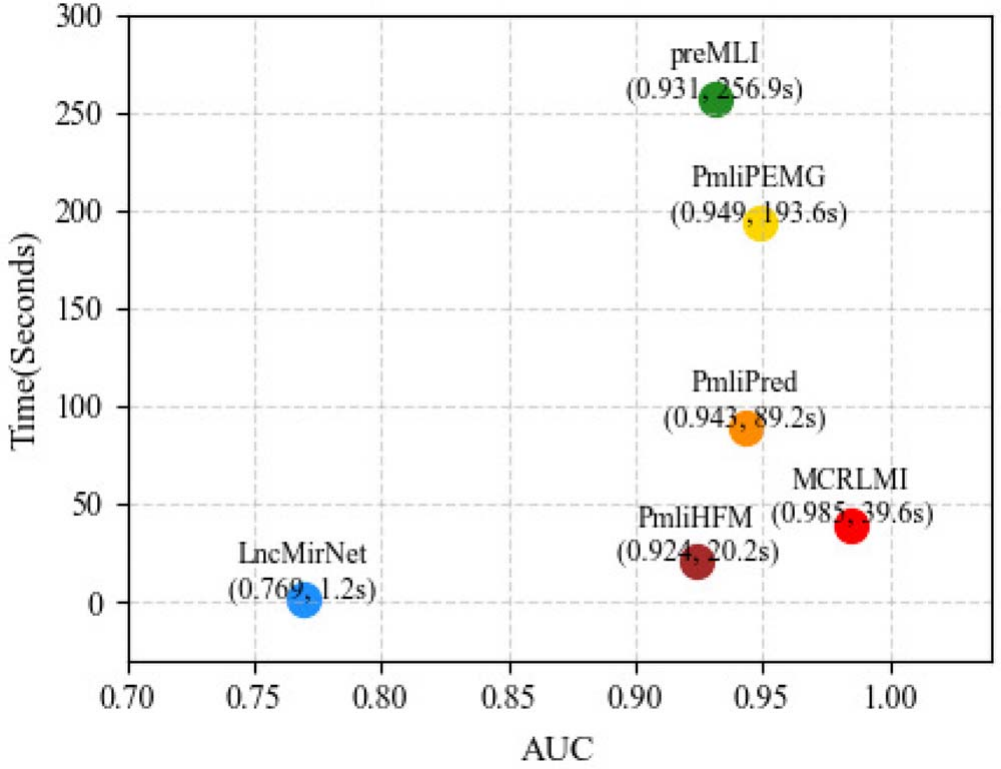
Time complexity analysis.

To provide an intuitive analysis, we compared the per-epoch training time of each model discussed in Section 3.2 and visualized the results in Fig. 7, where the x-axis represents AUC and the y-axis represents the per-epoch training time. Models located closer to the bottom-right corner of the figure generally perform better, indicating that they achieve higher AUC while requiring less training time. Since RNAI-FRID is a traditional machine learning model without epoch-based training, it was excluded from this comparison.

## Conclusion

This paper proposes a novel method based on multimodal contrastive learning to predict lncRNA-miRNA interactions, shorted for MCRLMI. MCRLMI integrates multimodal data to construct similarity and encoding matrices, utilizing GCN to capture local features and Transformer to extract global features. A multichannel attention mechanism is then applied for cross-modal fusion of the feature matrices. Next, contrastive learning is used to refine the embeddings of lncRNAs and miRNAs, and KAN generates the final predictions by appropriately incorporating these embeddings.

To assess its effectiveness, MCRLMI undergoes a comprehensive evaluation through a series of experiments on benchmark datasets. First, MCRLMI is compared with existing methods via cross-validation on both balanced and imbalanced datasets, as well as in hot and cold start scenarios. The results show that our method outperforms others, particularly in terms of robustness. Next, ablation studies confirm that each component of MCRLMI is essential, and their thoughtful integration is what sets MCRLMI apart from other methods. We also conduct parameter sensitivity analysis and visualize the embedding learning process to better understand its success. Additionally, case studies are performed to evaluate the practicality of MCRLMI. Overall, the extensive experimental results demonstrate that MCRLMI is highly effective in predicting LMIs and superior to existing models in the field.

In the future, we plan to develop a robust negative sample selection strategy to minimize its impact on prediction performance and further optimize the model for similar bioinformatics applications.

**Key Points**

- MCRLMI employs multiscale k-mers as input to the Transformer to capture long-distance dependency features, while multimodal similarity matrices are input into the GCN to extract local neighborhood structural features.
- The representative and discriminative features of lncRNAs and miRNAs are learned by contrastive learning with multichannel attention mechanisms from different modalities.
- Unlike straightforward embedding concatenation, KAN effectively characterizes lncRNA-miRNA interactions through its dynamic edge-function mapping mechanism.
- Extensive experiments demonstrate that MCRLMI outperforms existing approaches, with case studies further showcasing its ability to uncover potential lncRNA-miRNA interactions.

## Supplementary Material

Supplementary_file_S1_bbaf281

## References

[1] Yang S, Wang Y, Zhang S. et al. Ncresnet: Noncoding ribonucleic acid prediction based on a deep resident network of ribonucleic acid sequences. *Front Genet* 2020;11:90. 10.3389/fgene.2020.00090 32180792 PMC7059790

[2] Huang J-Z, Min Chen DE, Chen X-CG. et al. A peptide encoded by a putative lncrna hoxb-as3 suppresses colon cancer growth. *Mol Cell* 2017;68:171–184.e6. 10.1016/j.molcel.2017.09.015 28985503

[3] Cui J, Luan Y, Jiang N. et al. Comparative transcriptome analysis between resistant and susceptible tomato allows the identification of lnc rna 16397 conferring resistance to phytophthora infestans by co-expressing glutaredoxin. *Plant J* 2017;89:577–89. 10.1111/tpj.13408 27801966

[4] Liu X-Q, Li B-X, Zeng G-R. et al. Prediction of long non-coding rnas based on deep learning. *Genes* 2019;10:273. 10.3390/genes10040273 30987229 PMC6523782

[5] Wang W, Zhang L, Sun J. et al. Predicting the potential human lncrna–mirna interactions based on graph convolution network with conditional random field. *Brief Bioinform* 2022;23. 10.1093/bib/bbac463 36305458

[6] Peng W-X, Koirala P, Mo Y-Y. Lncrna-mediated regulation of cell signaling in cancer. *Oncogene* 2017;36:5661–7. 10.1038/onc.2017.184 28604750 PMC6450570

[7] Huang Z-A, Huang Y-A, You Z-H. et al. Novel link prediction for large-scale mirna-lncrna interaction network in a bipartite graph. *BMC Med Genomics* 2018;11:17–27. 10.1186/s12920-018-0429-8 30598112 PMC6311942

[8] Tsang FHC, Au SLK, Wei L. et al. Long non-coding rna hottip is frequently up-regulated in hepatocellular carcinoma and is targeted by tumour suppressive mir-125b. *Liver Int* 2015;35:1597–606. 10.1111/liv.12746 25424744

[9] Cantile M, Di Bonito M, De Bellis MT. et al. Functional interaction among lncrna hotair and micrornas in cancer and other human diseases. *Cancers* 2021;13:570. 10.3390/cancers13030570 33540611 PMC7867281

[10] Zhang H, Wang Y, Pan Z. et al. Ncrnainter: A novel strategy based on graph neural network to discover interactions between lncrna and mirna. *Brief Bioinform* 2022;23:bbac411. 10.1093/bib/bbac411 36198065

[11] Xinyu Y, Jiang L, Jin S. et al. Premli: A pre-trained method to uncover microrna–lncrna potential interactions. *Brief Bioinform* 2022;23:bbab470. 10.1093/bib/bbab470 34850810

[12] Tang Q, Nie F, Zhao Q. et al. A merged molecular representation deep learning method for blood–brain barrier permeability prediction. *Brief Bioinform* 2022;23:bbac357. 10.1093/bib/bbac357 36002937

[13] Fang Y, Pan X, Shen H-B. Recent deep learning methodology development for rna–rna interaction prediction. *Symmetry* 2022;14:1302. 10.3390/sym14071302

[14] Liu B, Fang L, Wang S. et al. Identification of microrna precursor with the degenerate k-tuple or kmer strategy. *J Theor Biol* 2015;385:153–9. 10.1016/j.jtbi.2015.08.025 26362104

[15] Tong X, Liu S. Cppred: Coding potential prediction based on the global description of rna sequence. *Nucleic Acids Res* 2019;47:e43–3. 10.1093/nar/gkz087 30753596 PMC6486542

[16] Lau JH, Baldwin T. An empirical evaluation of doc2vec with practical insights into document embedding generation. arxiv 2016. *arXiv preprint arXiv:1607.05368*

[17] Yang S, Yan Wang Y, Lin DS. et al. Lncmirnet: Predicting lncrna–mirna interaction based on deep learning of ribonucleic acid sequences. *Molecules* 2020;25:4372. 10.3390/molecules25194372 32977679 PMC7583909

[18] Ahmed NK, Rossi RA, Lee JB. et al. Role-based graph embeddings. *IEEE Trans Knowl Data Eng* 2020;34:2401–15. 10.1109/TKDE.2020.3006475

[19] Kang Q, Meng J, Cui J. et al. Pmlipred: A method based on hybrid model and fuzzy decision for plant mirna–lncrna interaction prediction. *Bioinformatics* 2020;36:2986–92. 10.1093/bioinformatics/btaa074 32087005

[20] Zhang P, Meng J, Luan Y. et al. Plant mirna–lncrna interaction prediction with the ensemble of cnn and indrnn. *Interdisciplinary Sciences: Computational Life Sciences* 2020;12:82–9. 10.1007/s12539-019-00351-w 31811618

[21] Kang Q, Meng J, Shi W. et al. Ensemble deep learning based on multi-level information enhancement and greedy fuzzy decision for plant mirna–lncrna interaction prediction. *Interdisciplinary Sciences: Computational Life Sciences* 2021;13:603–14. 10.1007/s12539-021-00434-7 33900552

[22] Kang Q, Meng J, Luan Y. Rnai-frid: Novel feature representation method with information enhancement and dimension reduction for rna–rna interaction. *Brief Bioinform* 2022;23:bbac107. 10.1093/bib/bbac107 35352114

[23] Wang Z, Liang S, Liu S. et al. Sequence pre-training-based graph neural network for predicting lncrna-mirna associations. *Brief Bioinform* 2023;24. 10.1093/bib/bbad317 37651605

[24] Chen L, Sun Z-L. Pmlihfm: Predicting plant mirna-lncrna interactions with hybrid feature mining network. *Interdisciplinary Sciences: Computational Life Sciences* 2023;15:44–54. 36223068 10.1007/s12539-022-00540-0

[25] Zhang L, Yang P, Feng H. et al. Using network distance analysis to predict lncrna–mirna interactions. *Interdisciplinary sciences: computational life sciences* 2021;13:535–45. 10.1007/s12539-021-00458-z 34232474

[26] Song J, Tian S, Long Y. et al. Islmi: Predicting lncrna-mirna interactions based on information injection and second-order graph convolution network. *IEEE/ACM Trans Comput Biol Bioinform* 2022;20:1737–45. 10.1109/TCBB.2022.3215151 36251906

[27] Zhao Z-Y, Lin J, Wang Z. et al. Sebglma: Semantic embedded bipartite graph network for predicting lncrna-mirna associations. *International Journal of Intelligent Systems* 2023;2023:2785436. 10.1155/2023/2785436

[28] Wang M-N, You Z-H, Li L-P. et al. Gnmflmi: Graph regularized nonnegative matrix factorization for predicting lncrna-mirna interactions. *Ieee Access* 2020;8:37578–88. 10.1109/ACCESS.2020.2974349

[29] Cai D, He X, Han J. et al. Graph regularized nonnegative matrix factorization for data representation. *IEEE Trans Pattern Anal Mach Intell* 2010;33:1548–60. 10.1109/TPAMI.2010.231 21173440

[30] Li X, Cui G, Dong Y. Graph regularized non-negative low-rank matrix factorization for image clustering. *IEEE transactions on cybernetics* 2016;47:3840–53. 10.1109/TCYB.2016.2585355 27448379

[31] Wang W, Chen H. Predicting mirna-disease associations based on lncrna–mirna interactions and graph convolution networks. *Brief Bioinform* 2023;24. 10.1093/bib/bbac495 36526276

[32] Zhang L, Ouyang C, Liu Y. et al. Multimodal contrastive representation learning for drug-target binding affinity prediction. *Methods* 2023;220:126–33. 10.1016/j.ymeth.2023.11.005 37952703

[33] Liu Z, Wang Y, Vaidya S. et al. Kan: Kolmogorov-Arnold networks arXiv preprint arXiv:2404.19756. 2024.

[34] Zhang L, Liu T, Chen H. et al. Predicting lncrna–mirna interactions based on interactome network and graphlet interaction. *Genomics* 2021;113:874–80. 10.1016/j.ygeno.2021.02.002 33588070

[35] Frankish A, Diekhans M, Jungreis I. et al. *Nucleic Acids Res* 2021;49:D916–23. 10.1093/nar/gkaa1087 33270111 PMC7778937

[36] Volders P-J, Anckaert J, Verheggen K. et al. Lncipedia 5: Towards a reference set of human long non-coding rnas. *Nucleic Acids Res* 2019;47:D135–9. 10.1093/nar/gky1031 30371849 PMC6323963

[37] Kozomara A, Birgaoanu M, Griffiths-Jones S. Mirbase: From microrna sequences to function. *Nucleic Acids Res* 2019;47:D155–62. 10.1093/nar/gky1141 30423142 PMC6323917

[38] Dechao B, Kuntao Y, Sun S. et al. Noncode v3. 0: Integrative annotation of long noncoding rnas. *Nucleic Acids Res* 2012;40:D210–5. 22135294 10.1093/nar/gkr1175 PMC3245065

[39] Miao Y-R, Liu W, Zhang Q. et al. lncrnasnp2: An updated database of functional snps and mutations in human and mouse lncrnas. *Nucleic Acids Res* 2018;46:D276–80. 10.1093/nar/gkx1004 29077939 PMC5753387

[40] Li H, Bin W, Sun M. et al. Multi-view graph neural network with cascaded attention for lncrna-mirna interaction prediction. *Knowledge-Based Systems* 2023;268:110492. 10.1016/j.knosys.2023.110492

[41] Wei J, He J, Chen K. et al. Collaborative filtering and deep learning based recommendation system for cold start items. *Expert Systems with Applications* 2017;69:29–39. 10.1016/j.eswa.2016.09.040

[42] Van der Maaten L, Hinton G. Visualizing data using t-sne. *Journal of machine learning research* 2008;9.

[43] Rousseeuw PJ . Silhouettes: A graphical aid to the interpretation and validation of cluster analysis. *Journal of computational and applied mathematics* 1987;20:53–65. 10.1016/0377-0427(87)90125-7

[44] Caliński T, Harabasz J. A dendrite method for cluster analysis. *Communications in Statistics-theory and Methods* 1974;3:1–27. 10.1080/03610927408827101

[45] Zhou X, Xiaohui X, Gao C. et al. Xist promote the proliferation and migration of non-small cell lung cancer cells via sponging mir-16 and regulating cdk8 expression. *American journal of translational research* 2019;11:6196–206. 31632587 PMC6789229

[46] Shi X, Sheng W, Jia C. et al. Hsa-mir-590-3p promotes the malignancy progression of pancreatic ductal carcinoma by inhibiting the expression of p27 and ppp2r2a via g1/s cell cycle pathway. *Onco Targets Ther* 2020;13:11045–58. 10.2147/OTT.S260499 33149617 PMC7605676

[47] Du B, Wang T, Yang X. et al. Sox9, mir-495, mir-590-3p, and mir-320d were identified as chemoradiotherapy-sensitive genes and mirnas in colorectal cancer patients based on a microarray dataset. *Neoplasma* 2019;66:8–19. 10.4149/neo_2018_170324N214 30509082

