# Supplementary material for: Enhancing LncRNA-miRNA interaction prediction with multimodal contrastive representation learning
Source: Brief Bioinform. 2025 Jun 17;26(3):bbaf281. doi: 10.1093/bib/bbaf281 (PMC12199918; doi:10.1093/bib/bbaf281)
Supplement: Supplementary_file_S1_bbaf281 [file supplementary_file_s1_bbaf281.docx]

**Supplementary Materials**

**A The calculation of sequence similarity**

The sequence similarity between lncRNAs ($LS$) is computed using Equation (1), where $L_{i}$ and $L_{j}$ represent the sequence lengths of the *i*-th and *j*-th lncRNA, respectively, and $X_{i}$ and $X_{j}$ represent the *i*-th and *j*-th lncRNA sequences. $\mathrm{Dlev}\left( X_{i},X_{j} \right)$ refers to the Levenshtein distance between the *i*-th and *j*-th lncRNA, which indicates the minimum number of single-character edit operations (insertion, deletion, or substitution) required to convert one sequence into the other. This distance serves as a measure of similarity between the two sequences.

$\text{LS}\left(X_{i},X_{j}\right)=\frac{\max\left(L_{i},L_{j}\right)-\mathrm{Dlev}\left(X_{i},X_{j}\right)}{\max\left(L_{i}-L_{j}\right)}$ (1)

The value of $LS$ is within the range of 0 to 1. A result closer to 1 means greater similarity between both. Similarly, the sequence similarity between miRNAs ($MS$) is calculated using the method above.

**B The calculation of expression profile similarity**

The expression profile similarity between lncRNAs ($LE$) is calculated using Equation (2), where $p_{i}^{t}$ and $p_{j}^{t}$ represent the *t-*th attribute value of the expression profiles of the *i*-th and *j*-th lncRNAs, respectively. $T$ denotes the number of relevant attributes. $p_{i}$ and $p_{j}$ represent the average values of the expression profiles of the *i*-th and *j*-th lncRNAs.

 (2)

The value of $LE$ is within the range of 0 to 1. A result closer to 1 means greater similarity between the expression profiles of the two lncRNAs. Similarly, the expression profile similarity between miRNAs ($ME$) is calculated using the method above.

**C The calculation of GIP kernel similarity**

We apply the Gaussian kernel function to the lncRNA-miRNA interaction network to calculate the GIP kernel similarity of lncRNAs and miRNAs. We define the adjacency matrix $IM\epsilon R^{U\times V}$, where $U$ and $V$ represent the number of lncRNAs and miRNAs, respectively. The GIP kernel similarity matrix $LG$ between each lncRNA pair $l_{i}$ and $l_{i}$ can be calculated using Equation (3), where $IP(l)$ denotes the interaction information between each lncRNA and all miRNAs which are obtained from the row of the adjacency matrix $IM$. $\lambda_{l}$ represents the bandwidth parameter. Similarly, the GIP kernel similarity between miRNAs (MG) is calculated using the method above.

 (3)

**D The p-nearest neighbor graph method**

Taking the lncRNA sequence similarity matrix $LS$ as an example, the weight matrix $G^{LS}$ is calculated using Equation (4). Here, $N_{i}^{p}$ and $N_{j}^{p}$ represent the p-nearest neighbor sets of the *i*-th and *j*-th lncRNAs, respectively. Based on $G^{LS}$, we obtain the sparse similarity matrix ${LS}^{*}=LS\odot G^{LS}$.

 (4)

Similarly, the sparse similarity matrix ${LE}^{*}$ for lncRNA expression profile similarity and the sparse similarity matrix ${LG}^{*}$ for lncRNA GIP kernel similarity are obtained. At the same time, the sparse similarity matrices ${MS}^{*}$, ${ME}^{*}$, ${MG}^{*}$ for miRNAs are also obtained.

**E Case study results and analysis**

We trained the model separately on the DS-R5 and DS-R10 datasets. Using the same test set as DS-R1, we performed prediction analysis on the same selected lncRNAs and miRNAs. Tables S1 and S2 present the top 20 prediction results on the DS-R5 dataset. Specifically, as shown in Table S1, 15 miRNAs associated with lncRNA NONHSAT137542.2 were validated in the lncRNASNP2 database. As shown in Table S2, 10 lncRNAs associated with miRNA hsa-miR-590-3p were validated. Similarly, Tables S3 and S4 show the top 20 prediction results on the DS-R10 dataset. As illustrated in Table S3, 16 miRNAs linked to lncRNA NONHSAT137542.2 were validated in the lncRNASNP2 database. As shown in Table S4, 12 lncRNAs associated with hsa-miR-590-3p were validated.

Compared with DS-R1, the model exhibited slightly reduced predictive performance on DS-R5 and DS-R10. This decline may be attributed to the increased proportion of negative samples, which introduces class imbalance. Such imbalance can cause training bias, making the model more inclined to learn features of the negative class, thereby increasing the number of false positives and false negatives and ultimately impairing the identification of positive interactions. Based on this analysis, we recommend using relatively balanced datasets in case studies to obtain more robust and reliable results.

In addition, we further analyzed the interactions predicted as ‘unknown’ by combining literature evidence and GO functional annotations. In DS-R5, hsa-miR-143-3p and hsa-miR-22-3p which were listed in Table S1 as ‘unknown’ predictions share the GO term GO:0060255 with the selected lncRNA NONHSAT137542.2. This term is involved in the regulation of macromolecule metabolic processes, including those related to proteins, nucleic acids, and polysaccharides. Likewise, NONHSAT028513.2 in Table S2 shares the same GO term with the selected miRNA hsa-miR-590-3p. In DS-R10, hsa-miR-145-5p and hsa-miR-26a-5p which were listed in Table S3 also share this functional annotation with NONHSAT137542.2. These findings suggest that, although these interactions have not yet been experimentally validated, their functional similarity indicates potential biological relevance, further highlighting the effectiveness and biological significance of our model in identifying potential functional associations.

Table S1. Validation of the top 20 miRNAs associated with NONHSAT137542.2 on DS-R5.

| Rank | miRNA | Evidence | Rank | miRNA | Evidence |
| --- | --- | --- | --- | --- | --- |
| 1 | hsa-miR-29c-3p | lncRNASNP2 | 11 | hsa-miR-22-3p | unknown |
| 2 | hsa-miR-374a-5p | lncRNASNP2 | 12 | hsa-miR-33a-5p | unknown |
| 3 | hsa-miR-18b-5p | lncRNASNP2 | 13 | hsa-miR-342-3p | lncRNASNP2 |
| 4 | hsa-miR-155-5p | lncRNASNP2 | 14 | hsa-miR-125a-5p | lncRNASNP2 |
| 5 | hsa-miR-18a-5p | lncRNASNP2 | 15 | hsa-miR-136-5p | lncRNASNP2 |
| 6 | hsa-miR-143-3p | unknown | 16 | hsa-miR-874-3p | unknown |
| 7 | hsa-miR-148b-3p | lncRNASNP2 | 17 | hsa-miR-329-3p | lncRNASNP2 |
| 8 | hsa-miR-33b-5p | unknown | 18 | hsa-miR-219a-5p | lncRNASNP2 |
| 9 | hsa-miR-15a-5p | lncRNASNP2 | 19 | hsa-miR-192-5p | lncRNASNP2 |
| 10 | hsa-miR-144-3p | lncRNASNP2 | 20 | hsa-miR-454-3p | lncRNASNP2 |

Table S2. Validation of the top 20 lncRNAs associated with hsa-miR-590-3p on DS-R5.

| Rank | lncRNA | Evidence | Rank | lncRNA | Evidence |
| --- | --- | --- | --- | --- | --- |
| 1 | NONHSAT078103.2 | unknown | 11 | NONHSAT075769.2 | unknown |
| 2 | NONHSAT102657.2 | unknown | 12 | NONHSAT127658.2 | unknown |
| 3 | NONHSAT007683.2 | lncRNASNP2 | 13 | NONHSAT080209.2 | lncRNASNP2 |
| 4 | NONHSAT113484.2 | unknown | 14 | NONHSAT007688.2 | lncRNASNP2 |
| 5 | NONHSAT007668.2 | lncRNASNP2 | 15 | NONHSAT113469.2 | unknown |
| 6 | NONHSAT056112.2 | unknown | 16 | NONHSAT071457.2 | unknown |
| 7 | NONHSAT137542.2 | lncRNASNP2 | 17 | NONHSAT031465.2 | unknown |
| 8 | NONHSAT063991.2 | lncRNASNP2 | 18 | NONHSAT028513.2 | unknown |
| 9 | NONHSAT137559.2 | lncRNASNP2 | 19 | NONHSAT007698.2 | lncRNASNP2 |
| 10 | NONHSAT007673.2 | lncRNASNP2 | 20 | NONHSAT034666.2 | lncRNASNP2 |

Table S3. Validation of the top 20 miRNAs associated with NONHSAT137542.2 on DS-R10.

| Rank | miRNA | Evidence | Rank | miRNA | Evidence |
| --- | --- | --- | --- | --- | --- |
| 1 | hsa-miR-152-3p | lncRNASNP2 | 11 | hsa-miR-1297 | unknown |
| 2 | hsa-miR-653-5p | lncRNASNP2 | 12 | hsa-miR-153-3p | lncRNASNP2 |
| 3 | hsa-miR-193b-3p | lncRNASNP2 | 13 | hsa-miR-195-5p | lncRNASNP2 |
| 4 | hsa-miR-543 | lncRNASNP2 | 14 | hsa-miR-379-5p | lncRNASNP2 |
| 5 | hsa-miR-15b-5p | lncRNASNP2 | 15 | hsa-miR-455-5p | unknown |
| 6 | hsa-miR-145-5p | unknown | 16 | hsa-miR-876-5p | lncRNASNP2 |
| 7 | hsa-miR-371a-5p | lncRNASNP2 | 17 | hsa-miR-26a-5p | unknown |
| 8 | hsa-miR-181c-5p | lncRNASNP2 | 18 | hsa-miR-129-5p | lncRNASNP2 |
| 9 | hsa-miR-30c-5p | lncRNASNP2 | 19 | hsa-miR-4306 | lncRNASNP2 |
| 10 | hsa-miR-193a-3p | lncRNASNP2 | 20 | hsa-miR-27a-3p | lncRNASNP2 |

Table S4. Validation of the top 20 lncRNAs associated with hsa-miR-590-3p on DS-R10.

| Rank | lncRNA | Evidence | Rank | lncRNA | Evidence |
| --- | --- | --- | --- | --- | --- |
| 1 | NONHSAT075769.2 | unknown | 11 | NONHSAT063991.2 | lncRNASNP2 |
| 2 | NONHSAT137559.2 | lncRNASNP2 | 12 | NONHSAT102616.2 | unknown |
| 3 | NONHSAT034667.2 | lncRNASNP2 | 13 | NONHSAT063968.2 | lncRNASNP2 |
| 4 | NONHSAT007684.2 | lncRNASNP2 | 14 | NONHSAT080207.2 | lncRNASNP2 |
| 5 | NONHSAT101254.2 | unknown | 15 | NONHSAT137542.2 | lncRNASNP2 |
| 6 | NONHSAT007695.2 | lncRNASNP2 | 16 | NONHSAT119908.2 | unknown |
| 7 | NONHSAT108634.2 | unknown | 17 | NONHSAT119678.2 | unknown |
| 8 | NONHSAT015410.2 | unknown | 18 | NONHSAT021834.2 | lncRNASNP2 |
| 9 | NONHSAT001975.2 | unknown | 19 | NONHSAT021844.2 | lncRNASNP2 |
| 10 | NONHSAT080205.2 | lncRNASNP2 | 20 | NONHSAT007667.2 | lncRNASNP2 |
